# Low glutamate diet improves working memory and contributes to altering BOLD response and functional connectivity within working memory networks in Gulf War Illness

**DOI:** 10.1038/s41598-022-21837-6

**Published:** 2022-10-26

**Authors:** Mackenzie T. Langan, Anna E. Kirkland, Laura C. Rice, Veronica C. Mucciarone, James Baraniuk, Ashley VanMeter, Kathleen F. Holton

**Affiliations:** 1 Department of Neuroscience, American University, Washington, DC USA; 2 Department of Psychiatry and Behavioral Sciences, Medical University of South Carolina, Charleston, SC USA; 3 Department of Neurology, Center for Functional and Molecular Imaging, Georgetown University, Washington, DC USA; 4 Department of Medicine, Georgetown University, Washington, DC USA; 5 Department of Health Studies, American University, Washington, DC USA; 6 Center for Neuroscience and Behavior, American University, Washington, DC USA; 7 Nutritional Neuroscience Lab, American University, 4400 Massachusetts Ave NW, Washington, DC 20016 USA

**Keywords:** Chronic pain, Neurological disorders

## Abstract

Gulf War Illness is a chronic multi-symptom disorder with severe cognitive impairments which may be related to glutamate excitotoxicity and central nervous system dysfunction. The low glutamate diet has been proposed as a comprehensive intervention for Gulf War Illness. We examined the effects of the low glutamate diet on verbal working memory using a fMRI N-back task. Accuracy, whole-brain blood oxygen level dependency (BOLD) response, and task-based functional connectivity were assessed at baseline and after 1 month on the diet (N = 24). Multi-voxel pattern analysis identified regions of whole-brain BOLD pattern differences after the diet to be used as seeds for subsequent seed-to-voxel functional connectivity analyses. Verbal working memory accuracy improved after the diet (+ 13%; *p* = 0.006). Whole-brain BOLD signal changes were observed, revealing lower activation within regions of the frontoparietal network and default mode network after the low glutamate diet. Multi-voxel pattern analysis resulted in 3 clusters comprising parts of the frontoparietal network (clusters 1 and 2) and ventral attention network (cluster 3). The seed-to-voxel analyses identified significant functional connectivity changes post-diet for clusters 1 and 2 (peak *p* < 0.001, cluster FDR *p* < 0.05). Relative to baseline, clusters 1 and 2 had decreased functional connectivity with regions in the ventral attention and somatomotor networks. Cluster 2 also had increased functional connectivity with regions of the default mode and frontoparietal networks. These findings suggest that among veterans with Gulf War Illness, the low glutamate diet improves verbal working memory accuracy, alters BOLD response, and alters functional connectivity within two networks central to working memory.

## Introduction

Gulf War Illness (GWI) is a chronic multisymptom disorder that affects 25–32% of 697,000 veterans deployed during the 1990–1991 Gulf War^1,2^. GWI is characterized by extreme fatigue, widespread chronic pain, gastrointestinal problems, and cognitive dysfunction^3^. Cognitive impairments in GWI include problems with learning, memory, concentration, and information processing^4^. These symptoms may be a consequence of exposure to neurotoxins during deployment, namely sarin and cyclo-sarin gases, pesticides, pyridostigmine bromide pills, oil well fires, and depleted uranium^5–7^.

Sarin gas, pesticides, and pyridostigmine bromide can cause permeability of the blood–brain barrier (BBB) and can directly inhibit acetylcholinesterase (AChE), an enzyme in the peripheral and central nervous system (CNS) responsible for the breakdown of acetylcholine (ACh) in the synaptic cleft^8^. Increased ACh causes enhanced CNS excitability with downstream effects on glutamatergic neurotransmission^9^. Excess glutamate can overexcite neurons, resulting in excitotoxicity^9–11^. Thus, symptoms of GWI may be caused by dysregulation of glutamatergic neurotransmission and excitotoxicity^12,13^.

We recently demonstrated that the low glutamate diet may be a novel, comprehensive intervention for GWI. Previous work has shown that 1-month on the low glutamate diet robustly reduced symptoms of fibromyalgia and irritable bowel syndrome, two disorders with highly overlapping symptomology as GWI^14^. This dietary intervention removes free (i.e., not bound to a protein) excitatory dietary amino acids (e.g., glutamate and aspartate) which could affect glutamatergic neurotransmission in the brain if an individual has an impaired BBB (a known consequence of stress and neurotoxic exposure)^15^ through inducing or perpetuating excitotoxicity, leading to oxidative stress, neuroinflammation, and cell death^13,14^. Glutamatergic dysregulation is associated with cognitive dysfunction and memory impairments^16,17^—hallmark symptoms of GWI^4,6,18^. Therefore, regulating glutamatergic neurotransmission with the low glutamate diet may result in improved cognitive functioning. Indeed, previous research by our lab demonstrated that after 1 month on the diet, veterans self-reported significantly improved cognitive symptoms, including improvements in self-reported memory and attention^19^. Objective cognitive testing with CNS Vital Signs (CNSVS) software also demonstrated improvements in cognitive functioning (general cognitive improvements, as well as psychomotor speed, processing speed, motor speed, executive functioning, cognitive flexibility, and reaction time); however, significant improvements in tests of visual and verbal memory recall were not observed^20^.

The objective of this study was to examine the effects of the low glutamate diet on working memory (WM), assessed using a verbal N-back task and functional magnetic resonance imaging (fMRI), which allows assessment of behavioral (accuracy) and neurobehavioral (blood oxygen-level dependent or BOLD signal) outcomes, as well as task-based functional connectivity (FC).

## Methods

Forty Gulf War veterans meeting both Kansas criteria^21^ and Center for Disease Control and Prevention (CDC) criteria^2^ for GWI were recruited from across the United States for a randomized placebo-controlled crossover clinical trial assessing the low glutamate diet as an intervention for GWI (NCT03342482; Registered November 17, 2017). This study was approved by the Institutional Review Boards at American University (IRB#2017-301) and Georgetown University (IRB# 2017-0811), as well as by the Human Research Protection Office (HRPO) of the US Army Medical Research and Materiel Command (HRPO Log Number A-20203.a). All participants provided written informed consent and all methods were performed in accordance with relevant guidelines and regulations.

**Figure 1:**
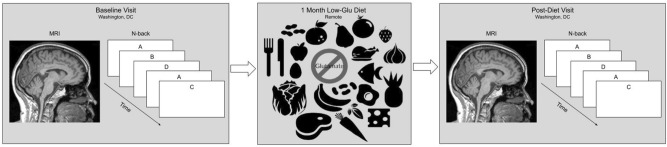
Neuroimaging study protocol. Participants came to Washington, DC for a baseline neuroimaging assessment, which included structural and functional (verbal N-back) MRI. Participants then completed detailed dietary training before being on the low glutamate diet for 1-month and returning for post-diet the structural and functional MRI.

The data shown herein is from the within-subjects pre-to-post dietary intervention phase of the study before randomization (random sequence generation was done using SAS 9.4) to the crossover placebo-controlled challenge period which did not assess neuroimaging outcomes. The clinical trial sample size was powered based upon previous work in fibromyalgia^14^. Eligibility criteria has been outlined elsewhere^19^, but briefly, participants had to be < 75 years of age, willing to change their diet, without a substance use disorder in the last year, and not on medication which affects glutamatergic neurotransmission. MRI exclusions included presence of a non-MR compatible implant/device, extreme claustrophobia, or body size restrictions. Thirty participants were eligible for scanning, of which 25 participants completed the scanning protocol (n = 2 dropped out prior to diet initiation, n = 3 had incomplete scanning sessions, Fig. 1).

### Demographics and measures of memory and cognition

At baseline, and after the 1-month diet (post-diet assessment), anthropometrics were measured. A self-report excitotoxin food frequency questionnaire (FFQ) was used to estimate participants' consumption of excitotoxins, and this served as a measure of dietary compliance during the study. Participants also completed a symptom questionnaire, and computerized cognitive testing (CNSVS). The CNSVS results for the full GWI sample have been published elsewhere^20^.

### N-back working memory (WM) task

Subjects were trained on the verbal N-back task (Supplementary Fig. 1) and completed three practice rounds during a mock scanning session at each assessment to check for basic understanding and to equalize practice. Task specifics can be found in supplementary materials.

### Image acquisition

Data were acquired at Georgetown University’s Center for Functional and Molecular Imaging in Washington, DC. Structural and functional MRI data were acquired on a Siemens 3 T Magnetom Trio system scanner using a 12-element head coil array (n = 9). Halfway through the study, the scanner underwent a system upgrade, after which data were acquired on a Magnetom Prisma Fit system using a 20-element head coil (n = 15). The scanner upgrade had no effect on results and none of the subjects had their pre- versus post-intervention scans split between scanner versions. Structural 3D T1-weighted Magnetization Prepared Rapid Acquisition Gradient Echo (MPRAGE) image parameters were TR/TE = 1900/2.52 ms, flip angle = 9°, TI = 900 ms, FoV read = 250 mm, 176 slices, slice thickness = 1.0 mm, and voxel size = 1 × 1 × 1 mm^3^. fMRI data consisted of interleaved T2-weighted gradient echo-planar images (EPIs) acquired during the 5-min task. Parameters were TR/TE = 2500/30 ms, flip angle = 90°, FoV = 205 mm, 47 slices, slice thickness = 3.2 mm, and voxel size = 3.2 × 3.2 × 3.2 mm^3^ isotropic.

### The low glutamate diet

Following baseline assessment, all subjects received in-depth diet training before following the low glutamate diet for 1 month. The low glutamate diet is a whole food diet that restricts consumption of free glutamate and aspartate. These are mainly found as flavor-enhancing food additives (e.g., MSG, hydrolyzed protein, aspartame, etc.) but are also naturally occurring in some foods (e.g., soy sauce, fish sauces, aged cheeses, etc.). The diet excludes the consumption of excitotoxins and optimizes the consumption of antioxidants and nutrients which are protective against excitotoxicity. More information on the low glutamate diet can be found elsewhere^19^.

### Statistical methods

#### Demographics, cognitive functioning (CNSVS), and N-back behavioral data

Averages for BMI, FFQ, symptom score, and CNSVS cognitive functioning measures for each subject were calculated at baseline and post-diet. For the N-back, mean percentage accuracy was calculated separately for both conditions (0-back/2-back). Data normality was assessed using Shapiro–Wilk tests, and a paired t-test or Wilcoxon signed rank test was used for pre-post diet comparisons.

#### fMRI: whole-brain and functional connectivity (FC) analyses

Functional data from the 2-back portion of the WM task were analyzed in two ways to investigate changes associated with the low glutamate diet: (1) brain-wide BOLD signal, and (2) task-based FC. See supplementary materials for preprocessing methods. For both techniques, an outlier was defined as any volume > 2 SD from the mean for translational or rotational parameters (3 translation, 3 rotation, and their first-order derivatives). Subjects were excluded if > 20% of their total volumes met this criterion. One participant was removed before first-level analysis, resulting in 24 participants included in the final analyses.

#### Whole-brain BOLD signal analysis

Following preprocessing, mass-univariate analyses were conducted in SPM12. First-level analysis was performed using a one sample t-test with the contrasts 2-back > 0-back and 0-back > 2-back at baseline and post-diet. The resulting contrast maps for the contrast of interest, 2-back > 0-back, were then used in the second-level analysis using the factorial design specification with a two-tailed, paired t-test without replication over sessions to measure contrasts within subjects (Post Diet > Baseline and Baseline > Post Diet, voxel level *p* < 0.001, cluster level: FDR *p* < 0.05, k_E_ (extent threshold (voxels) ≥ 25).

#### Task-based functional connectivity processing and analysis

CONN Functional Connectivity Toolbox (19c)^22^ was used to assess whole-brain FC changes. For FC specifically, we used a multi-voxel pattern analysis (MVPA) as a data driven approach to find clusters of interest with differences in whole-brain FC (rather than a priori regions of interest) followed by seed-to-voxel connectivity assessment to determine the directionality of FC differences. This approach has been previously used to look at whole brain networks and resting/FC in several different populations and to assess treatment outcomes^23–30^.

MVPA summarizes the entire voxel-to-voxel connectome for each subject to delineate where brain activity differed before and after the dietary treatment. MVPA takes the residualised BOLD time series within each voxel to calculate connectivity with all other voxels in the brain and then uses principal component analysis to reduce the dimensionality of the data to 64 dimensions for each voxel. We used the first 5 components (recommended N/5 for reasonable sensitivity and power; accounting for 69.5% cumulative variance) for the final MVPA-derived maps used in the second-level analysis. For the second-level analysis, a paired, two-tailed t-test for the contrast 2-back post-diet v. 2-back pre-diet was run to identify brain activation clusters that had functional changes associated with the dietary intervention (voxel level: *p* < 0.001, cluster FDR *p* < 0.05, k_E_ ≥ 75). Post-hoc seed-to-voxel analyses were conducted to assess directionality of the functional changes.

The resulting clusters from the MVPA-derived connectivity maps were saved as regions-of-interest (seeds) to complete post-hoc seed-to-voxel analyses. The seed-to-voxel analyses used linear regression at the first level to create FC maps from each seed for each subject. The second-level analysis used a paired two-tailed t-test for the contrast 2-back post-diet vs. 2-back pre-diet (voxel level: *p* < 0.001, cluster FDR *p* < 0.05, k_E_ ≥ 75). The two-sided second-level tests provided positive (increased connectivity) and negative (decreased connectivity) test-static values to assess the directionality of FC changes between testing sessions. Significant clusters are described within functional networks^31,32^.

### Ethics approval

The Institutional Review Boards at American University and Georgetown University approved this study, in addition to the Human Research Protection Office (HRPO) of the US Army Medical Research and Materiel Command (HRPO Log NumberA-20203.a).

### Consent to participate

All participants provided written informed consent.

## Results

### Measures of memory and cognition

In alignment with the full sample results, there was an overall significant decrease in BMI (*p* = 0.016), FFQ (*p* < 0.001), and Symptom Score (*p* < 0.001). For CNSVS, the neuroimaging sample showed improved verbal memory (*p* = 0.046) and overall cognitive functioning measured by the neurocognitive index (*p* = 0.037) (Supplementary Table 1).

### N-back WM task: behavioral data

Accuracy (percent correct) on the 0-back test was high at both time points (pre-diet mean (SD) = 97% (3%); post-diet mean (SD) = 96% (6%); *p* > 0.05), demonstrating that subjects understood the task and followed instructions. Accuracy on the 2-back WM task significantly improved after 1 month on the low glutamate diet (+ 13% correct; pre-diet mean (SD) = 58% (26%), post-diet mean (SD) = 71% (22%); *p* = 0.006).

### Whole-brain BOLD signal findings

There were no significant differences in BOLD signal after the diet that survived correction for multiple comparisons (baseline > post-diet; FDR *p* > 0.05). Due to the small sample size, an exploratory analysis was run (*p* < 0.005, k > 25) which corresponded with reduced BOLD signal during the 2-back in several brain areas after the diet month (Supplementary Table 2).

### Functional connectivity findings

#### MVPA-derived clusters

**Figure 2:**
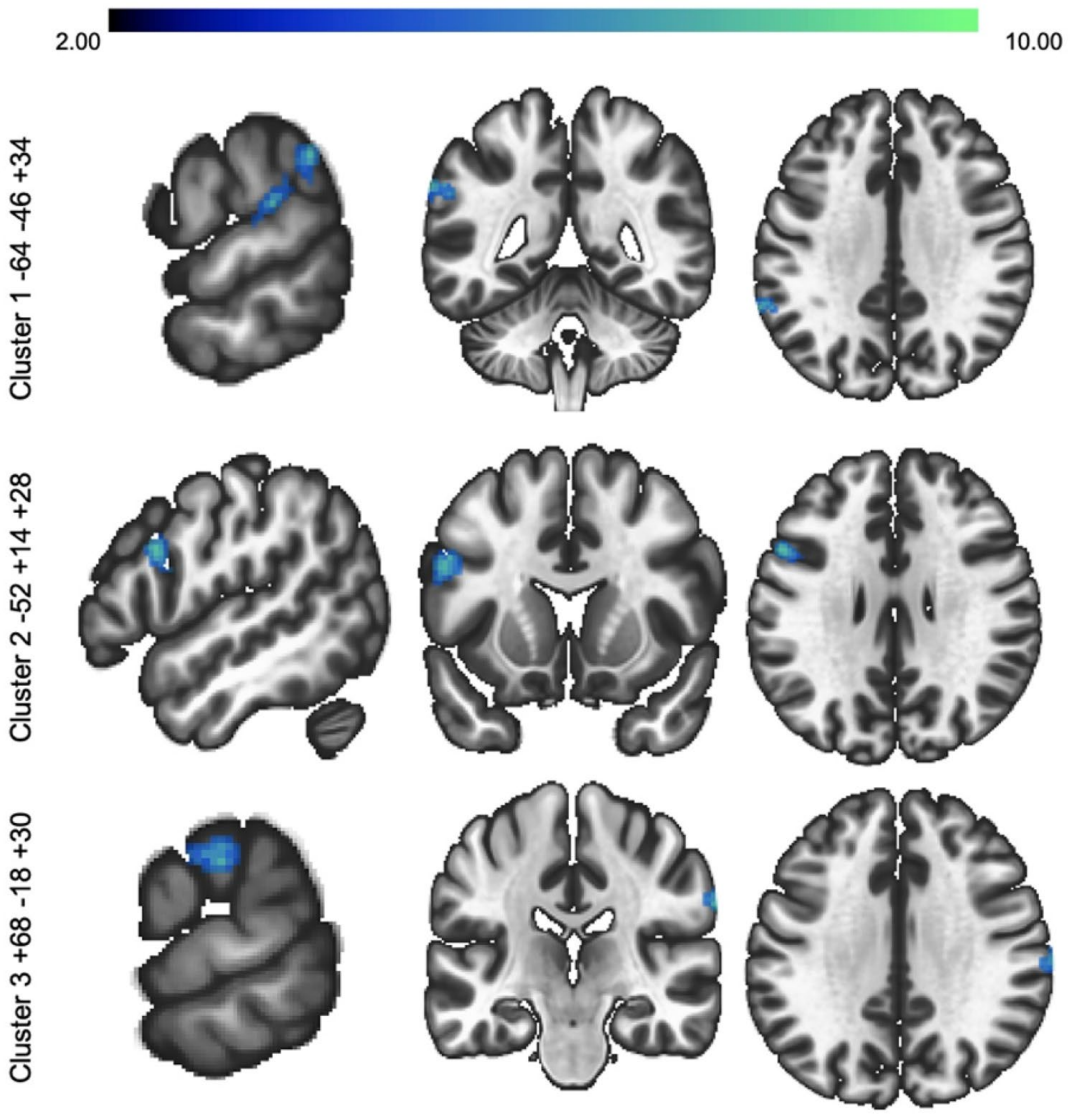
MVPA clusters. Three clusters were found to have significant differences after the 1-month diet (2-back post diet > 2-back baseline): (1) L. Posterior Supramarginal Gyrus; (2) L. Inferior Frontal Gyrus; and (3) R. Anterior Supramarginal Gyrus. MVPA analysis thresholding: voxel p < 0.001 (uncorrected), cluster p < 0.05 (FDR), k > 75.

MVPA analyses indicated that there were 3 significant clusters that had BOLD-related changes during the 2-back task after the 1-month diet (Table 1, Fig. 2). These clusters were used as seeds in the seed-to-voxel analysis described below.

**Table 1:** MVPA-derived clusters with significant differences during N-back task after the 1-month low glutamate diet (Post-diet _2-back_ > Baseline _2-back_).

	Main region	Additional region	MNI (X, Y, Z)	K	*F* (5, 19)	p-FDR	Cohen’s *d*	Network
Cluster 1	L. Posterior Supramarginal Gyrus	L. Parietal Operculum L. Anterior Supramarginal Gyrus L. Planum Temporale	− 64 − 46 + 34	140	6.97	< 0.0001	1.08	FPN
Cluster 2	L. Inferior Frontal Gyrus, pars opercularis	L. Middle Frontal Gyrus L. Precentral Gyrus	− 52 + 14 + 28	115	8.25	< 0.0001	1.17	FPN
Cluster 3	R. Anterior Supramarginal Gyrus	R. Postcentral Gyrus	+ 68 − 18 + 30	91	7.92	< 0.0001	1.15	VA

MVPA analysis thresholding: voxel p < 0.001 (uncorrected), cluster p < 0.05 (FDR), k > 75.

*Network* cortical and cerebellar networks from Yeo et al. 2011 and Buckner et al. 2011 7-network parcellations, *FPN* frontoparietal network, *VAN* ventral attention network.

#### Seed-to-voxel analysis of functional connectivity

The seed-to-voxel analysis assessed FC changes during the 2-back condition between the 3 seed regions (MVPA clusters) and the rest of the brain (Table 2).

**Table 2:** Task-based functional connectivity differences for MVPA-derived seeds after 1-month on the low glutamate diet (2-back post-diet > 2-back baseline).

MVPA seed	Main region	Additional region(s)	MNI (X, Y, Z)	K	*T* (df = 23)	p-FDR	Cohen’s *d*	Network
Seed 1	L. Central Opercular Cortex	L. Planum Temporal L. Heschl’s Gyrus L. Parietal Operculum Cortex L. Insular Cortex L. Posterior Superior Temporal Gyrus	− 46 − 16 + 16	254	− 6.79	0.0002	2.83	
	R. Superior Temporal Gyrus	R. Heschl’s Gyrus R. Planum Temporale R. Putamen R. Planum Polare R. Insula	+ 60 − 24 + 06	235	− 6.86	0.0002	2.86	SM
	L. Superior Temporal Gyrus	L. Temporal Pole L. Frontal Orbital Cortex L. Planum Polare L. Frontal Operculum Cortex L. Central Opercular Cortex L. Insula	− 48 + 06 − 10	221	− 5.91	0.0002	2.46	SM
	L. Putamen	L. Insular Cortex L. Central Opercular Cortex	− 34 + 00 + 06	132	− 7.29	0.0051	3.04	VAN
	L. Postcentral Gyrus	L. Anterior Supramarginal Gyrus	− 60 − 18 + 38	98	− 5.59	0.0181	2.33	VAN
	L. Inferior Frontal Gyrus, pars opercularis	L. Central Opercular Cortex L. Precentral Gyrus L. Planum Polare	− 52 + 08 + 10	92	− 5.14	0.0199	2.14	VAN
Seed 2	L Angular Gyrus	L. Inferior parietal Lobule	− 38 − 72 + 44	160	5.33	0.0031	2.22	DMN
	R. Cerebellum Crus I	R. Cerebellum Crus II R. Lingual Gyrus R. Lobule VI	+ 10 − 84 − 26	94	5.40	0.0358	2.25	FPN
	R. Supplementary Motor Cortex	L. Superior Frontal Gyrus R/L. Supplementary Motor Cortex R/L. Precentral Gyrus	+ 02 − 14 + 68	523	− 6.59	< 0.0001	2.75	SM
	R. Superior Parietal Lobule	R. Postcentral Gyrus	+ 20 − 50 + 66	292	− 6.17	< 0.0001	2.57	SM
	L. Paracentral Lobule	R/L. Middle Cingulum	− 04 − 34 + 54	81	− 4.77	0.0484	1.98	SM
	L. Precuneus	L. Superior Parietal Lobule	− 10 − 50 + 58	79	− 5.68	0.0484	2.36	VAN

*Network* cortical and cerebellar networks from Yeo et al. (2011) and Buckner et al. (2011) 7-network parcellations, *SC* subcortical, *SM* somatomotor, *VAN* ventral attention network, *DMN* default mode network, *FPN* frontoparietal network.

**Figure 3:**
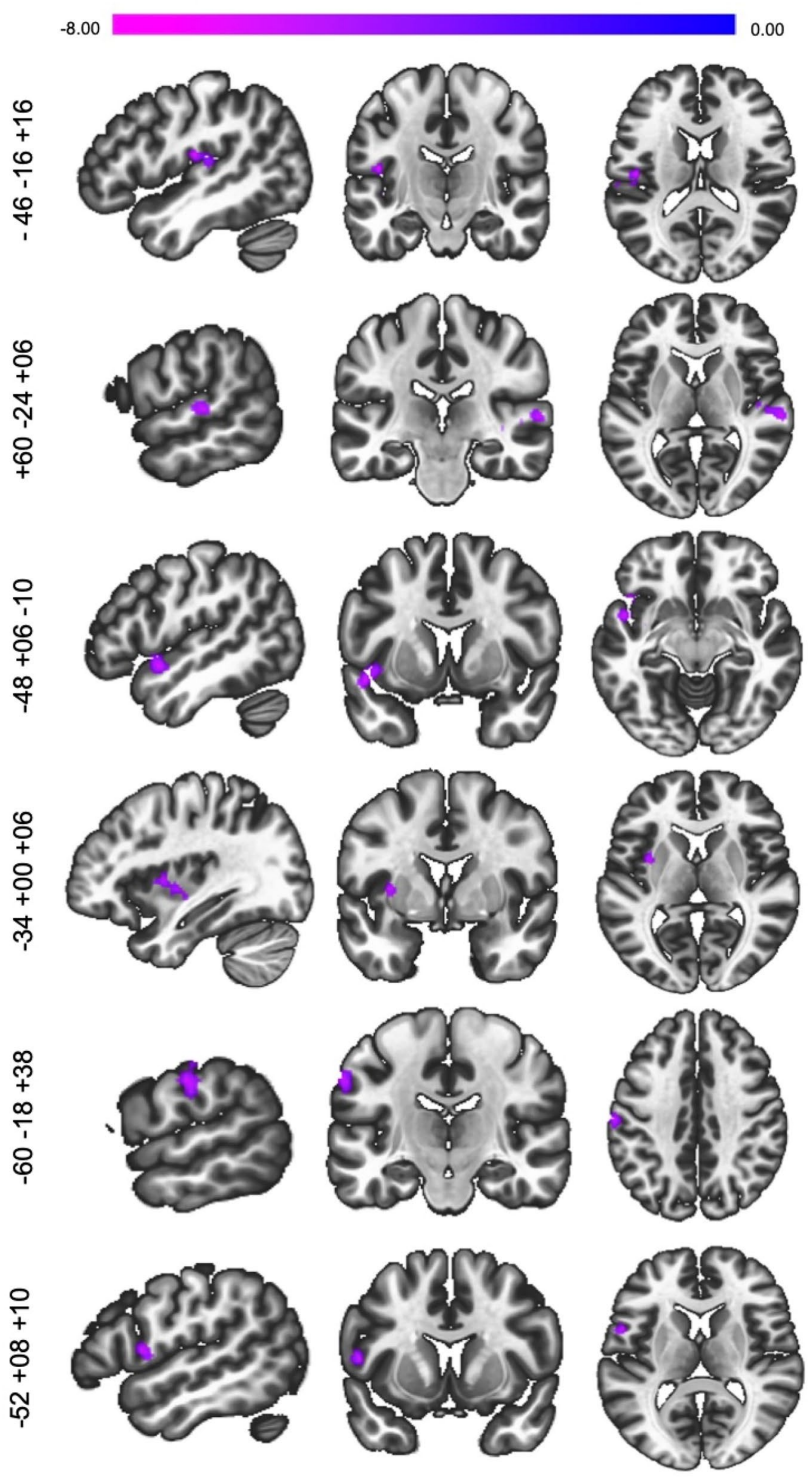
Seed-to-voxel functional connectivity changes at post-diet (2 back post diet > 2-back baseline) for Seed 1 (L. Posterior Supramarginal Gyrus) voxel level: *p* < 0.001, cluster FDR *p* < 0.05, k_E_ ≥ 75).

Overall, Seed 1 (frontoparietal network; FPN) had decreased connectivity with regions within the ventral attentional network (VAN; L. putamen, postcentral gyrus, inferior frontal gyrus), and somatomotor (SM; bilateral superior temporal gyrus) network (Fig. 3).

**Figure 4:**
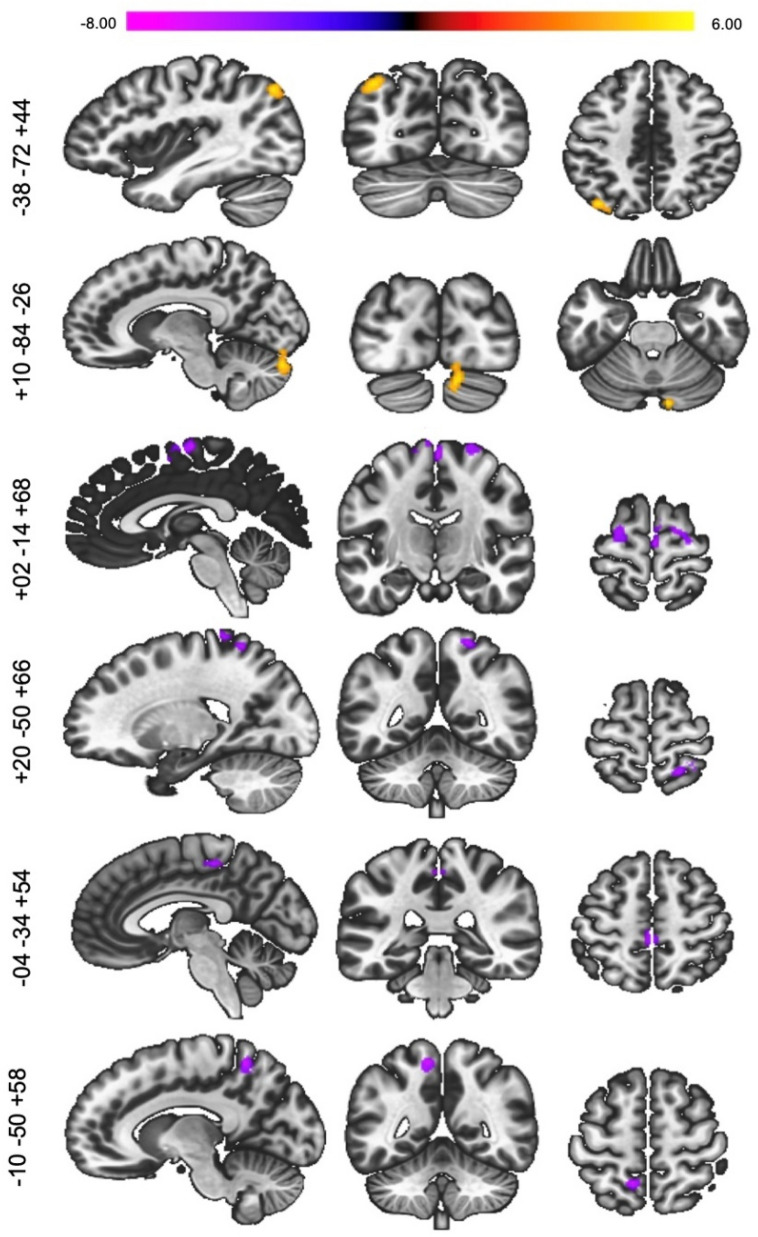
Seed-to-voxel functional connectivity changes at post-diet (2-back post diet > 2-back baseline) for Seed 2 (L. Inferior Frontal Gyrus) voxel level: *p* < 0.001, cluster FDR *p* < 0.05, k_E_ ≥ 75).

For Seed 2 (FPN), there was increased connectivity between regions within the default mode network (DMN; L angular gyrus) and another region within the FPN (R. cerebellum crus I/II), and decreased connectivity with regions within the SM (R. supplementary motor, superior parietal lobule, L. paracentral lobule) and VAN (L. precuneus; Fig. 4). There were no significant differences with Seed 3. Supplementary Fig. 2 displays an overview of FC changes.

## Discussion

The present study assessed the effects of the low glutamate diet on working memory (WM) and underlying neurological impairments in GWI, which may manifest as cognitive dysfunction among veterans with GWI. Our findings suggest that the diet is associated with improvements in verbal working memory (VWM) accuracy in GWI which may be due to functional connectivity (FC) changes within pertinent networks. Since this is the first time testing the low glutamate diet on VWM memory in GWI and the sample size is small, future research with greater power and more cognitive testing will help to determine the replicability of the behavioral and neural findings.

While there were no significant differences in whole-brain BOLD response, exploratory analyses at a less stringent threshold revealed decreased activation within task-relevant regions following the diet. Regions of decreased activation in BOLD response overlapped with significant clusters/seeds in the MVPA analysis (left inferior frontal gyrus and left middle frontal gyrus) and within the seed-to-voxel analysis (right cerebellar crus II), providing a source of consistency across analysis type.

The FC analysis resulted in several interesting findings. Following the diet, increased FC was observed within regions of the FPN and between the FPN and DMN, networks that are central to WM. Additionally, reduced FC was observed between the FPN and the sensory/attention reorienting networks (VAN and SM). Taken together, these findings suggest that increased FC within top-down networks relevant to WM and decreased FC within bottom-up sensorimotor networks may underlie the improvement in WM. Furthermore, these findings suggest that cognitive dysfunction and affected functional networks in GWI are malleable after dietary intervention.

Veterans with GWI consistently present with high activation in networks underlying cognitive tasks during fMRI studies, which suggests a higher cognitive load than healthy controls^33^. The reported BOLD and FC findings support this idea, as decreases in whole-brain BOLD activation and FC were noted after the diet, which may indicate an increased signal-to-noise ratio and less interference from sensory regions that corresponds with improvements on the VWM task. Specifically, the MVPA analysis found three clusters/seeds, each with relation to VWM^34,35^, that displayed significant changes post-diet: (1) left posterior supramarginal gyrus (FPN), (2) left inferior frontal gyrus (FPN), and (3) right anterior supramarginal gyrus (VAN).

Since MVPA does not provide directional information, we conducted a seed-to-voxel analysis to assess task-based FC during the verbal 2-back task. These findings provide insights into how the low glutamate diet may improve VWM in GWI. The FPN is a critical network recruited during WM and other cognitive tasks^36,37^, and all connectivity-related changes were found in relation to FPN seeds. For both seeds 1 and 2, we found decreased connectivity within regions composing the SM and VAN. The SM and VAN are networks associated with sensory-motor^32,38,39^ and reorienting of attention^40,41^, respectively. Since the VAN is a bottom-up attention orienting system, suppressed activation within this network is associated with better task performance^41^. The decreased connectivity between the FPN and sensory/motor/reorienting networks may be indicative of network restructuring and improved processing efficiency or less sensory interference, allowing for improved performance on the WM task. Additionally, whole-brain BOLD changes were mainly within regions associated with the FPN; thus, the decrease in activation at post-diet assessment within these regions may further indicate possible network reconstruction.

Like the FPN, the DMN is also known to underpin cognitive ability^42^. Previous work has shown dynamic, opposing BOLD signal response within these networks during cognitive tasks^43^, especially during tasks with high WM load^44,45^. However, current research is finding more nuance in how the FPN and DMN function in relation to each other, particularly during WM tasks. Recent findings have reported that the FPN directs FC during WM, and that sub-networks within the FPN may be anti-correlated (classical framework) or correlated with DMN activation during the task^46^. As such, our findings support the latter, as FC was strengthened between the FPN and DMN (left superior lateral occipital cortex). We also found strengthened connectivity within the FPN (left inferior frontal gyrus) to right cerebellar crus I/II/lobule VI, cerebellar regions which are part of the FPN and involved in cognitive and executive control^31,47–49^. Previous studies examining language and VWM have shown task-based activation of the right crus I, II, and lobule VI^35,50^. Further research is needed to parse out the relationship between the FPN and DMN, as well as within the FPN, and how these networks relate to WM in GWI.

This is the first study to test the effects of the low glutamate diet on VWM and uses advanced analyses to investigate task-based FC changes. As such, we were able to probe underlying mechanisms that may contribute to WM improvements found after the diet month. Additionally, we had very good dietary compliance and a wide range of symptom improvements, as previously reported^19,20,51^, providing confidence that the diet is a feasible and comprehensive intervention for GWI. Additionally, the regions and lateralization of significant clusters across the fMRI analyses matched with activation typically associated with VWM tasks^37,47,52–55^, providing confidence that the results are capturing task-based changes. Limitations of the study would include the small the number of subjects who completed MRI testing due to limitations such as presence of shrapnel and other ferromagnetic materials. An increased sample size may provide the ability to better understand how the diet may affect whole-brain BOLD signal response, as we did not find significant results at our a priori threshold level but did see indications of general decreases in post-diet BOLD response at a less stringent level. Additionally, this study focused on one assessment of working memory. We cannot rule out the possibility that practice effects are contributing to the significant improvements reported here; however, previous work in healthy populations found no significant differences on a VWM n-back tasks after even shorter treatment periods (2–14 days)^56–58^. A sample size of approximately 34 veterans with GWI would provide 85% power to detect differences in working memory with the given effect size^59^. Future studies should incorporate more robust and diverse cognitive tasks to corroborate and elaborate the current findings.

## Conclusion

The current study suggests that the low glutamate diet improves VWM accuracy, alters BOLD response, and produces FC changes within networks related to WM in veterans suffering from GWI. The FPN was highly implicated in task-based FC changes, supporting its central role in WM and cognitive functioning. Additionally, this study suggests that decreased connectivity with sensory-motor and reorienting systems, and increased connectivity with DMN and FPN may support improved WM in veterans with GWI. Further research is needed to replicate these findings in a larger group of veterans and to fully understand the effects of the diet within other networks related to cognition.

## Supplementary Information

Supplementary Information.

## Data Availability

The data that support the findings of this study are available from the corresponding author upon request.
