# Supplementary material for: Low glutamate diet improves working memory and contributes to altering BOLD response and functional connectivity within working memory networks in Gulf War Illness
Source: Sci Rep. 2022 Oct 26;12:18004. doi: 10.1038/s41598-022-21837-6 (PMC9606252; doi:10.1038/s41598-022-21837-6)
Supplement: Supplementary file 1 — Supplementary Information. [file 41598_2022_21837_MOESM1_ESM.docx]

Supplementary Material:

**Back Working Memory Task**

**
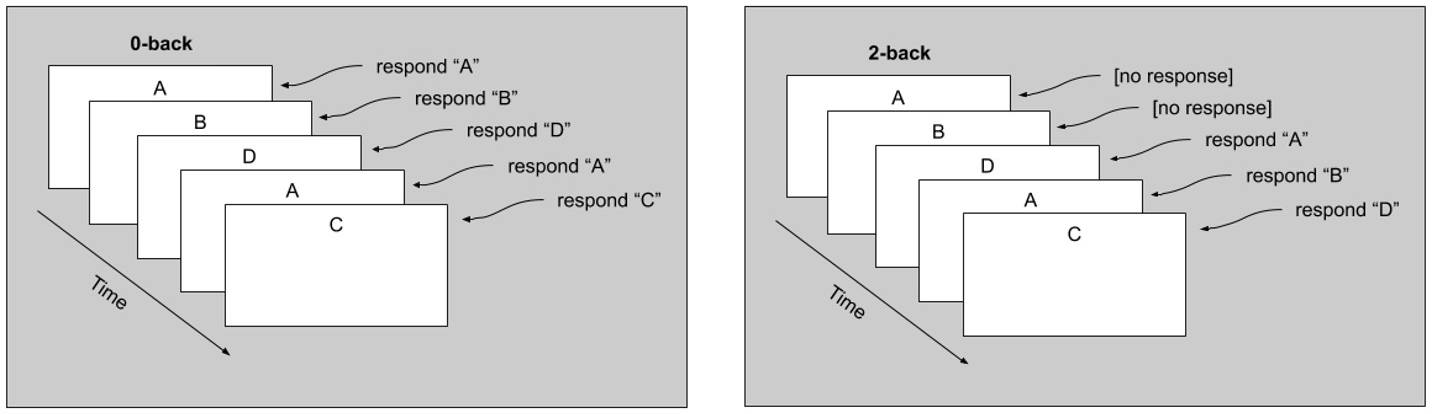
**

**Supplementary Figure 1.** Schematic example of the 0-back and 2-back blocks of the fMRI N-Back task.

During the task, subjects were presented with a 2-second instruction screen (rest, 0-back, 2-back) followed by a fixation cross (8 sec) before the alternating 0-back and 2-back stimulus blocks (18 sec) for five cycles (5 minutes). Each block consisted of 9 individual pseudo-randomly selected uppercase letters (A, B, C, D), displayed for 0.8 seconds, followed by an inter-stimulus interval of 1.2 seconds (blank screen) (resulting in 90 total trials across the 0-back and 2-back). For the 0-back blocks, subjects were presented with a single letter and asked to report the letter they were viewing on the screen with corresponding button presses. For 2-back blocks, subjects were asked to press the button corresponding to the letter two prior to the one presented on the screen. Responses were recorded using an MRI-compatible fibre-optic four-choice button box that was held with both hands, and buttons were pushed with pointer and middle fingers with the letter choices A, B, C, and D corresponding with the buttons from left to right.

***Whole-Brain BOLD Signal Processing***

Image analysis was performed using Statistical Parametric Mapping (SPM12, RRID:SCR_007037) and MATLAB and Statistics Toolbox Release 2021b (The MathWorks, Inc., Natick, Massachusetts, United States) to assess BOLD signal changes during the 2-back task at baseline and at post-diet assessment.

Imaging data were converted from DICOM to BIDS and were preprocessed using the FMRIPREP pipeline version stable (Esteban et al., 2019), a Nipype (Gorgolewski et al., 2011, Gorgolewski et al., 2018) based tool without FreeSurfer reconstruction. Each T1-weighted (T1w) volume was corrected for intensity non-uniformity using N4BiasFieldCorrection v2.1.0 (Tustison et al., 2010) and skull-stripped using antsBrainExtraction.sh v2.1.0 (using the OASIS template). Spatial normalization to the ICBM 152 Nonlinear Asymmetrical template version 2009c (Fonov et al., 2009) was performed through nonlinear registration with the antsRegistration tool of ANTs v2.1.0 (Avants et al., 2008), using brain-extracted versions of both T1w volume and template. Brain tissue segmentation of cerebrospinal fluid (CSF), white-matter (WM) and gray-matter (GM) was performed on the brain-extracted T1w using FAST (Zhang et al., 2001).

Functional data was slice-time corrected using 3dTshift from AFNI v16.2.07 (Cox and Hyde, 1997) and motion corrected using mcflirt (FSL v5.0.9 (Jenkinson et al., 2002)). This was followed by co-registration to the corresponding T1w image using boundary-based registration (Greve and Fischl, 2009) with six degrees of freedom, using flirt (FSL). Motion correcting transformations, BOLD-to-T1w transformation and T1w-to-template (MNI) warp were concatenated and applied in a single step using antsApplyTransforms (ANTs v2.1.0) and Lanczos interpolation (Lanczos, 1964).

Physiological noise regressors were extracted by applying CompCor (Behzadi et al., 2007). Principal components were estimated for the two CompCor variants: temporal (tCompCor) and anatomical (aCompCor). A mask to exclude signal with cortical origin was obtained by eroding the brain mask, ensuring it only contained subcortical structures. Six tCompCor components were then calculated including only the top 5% variable voxels within that subcortical mask. For aCompCor, six components were calculated within the intersection of the subcortical mask and the union of CSF and WM masks calculated in T1w space, after their projection to the native space of each functional run. Frame-wise displacement (Power et al., 2014) was calculated for each functional run using the implementation of Nipype. ICA-based Automatic Removal of Motion Artifacts (AROMA) was used to generate aggressive noise regressors as well as to create a variant of data that was non-aggressively denoised (Pruim et al., 2015). Many internal operations of FMRIPREP used Nilearn (Deschamps et al., 2014), principally within the BOLD-processing workflow. For more details of the pipeline see https://fmriprep.readthedocs.io/en/stable/workflows.html.

Following preprocessing, mass-univariate analyses were conducted in SPM12. First-level analysis was performed using a one sample t-test with the contrasts 2-back > 0-back and 0-back > 2-back at baseline and post-diet. The resulting contrast maps for the contrast of interest, 2-back > 0-back, were then used in the second-level analysis using the factorial design specification with a two-tailed, paired t-test without replication over sessions to measure contrasts within subjects (Post Diet > Baseline and Baseline > Post Diet, voxel level *p*<0.001, cluster level: FDR *p* < 0.05, k_E (_extent threshold (voxels) ≥25).

***Task-Based Functional Connectivity Processing***

The same outlier criterion was used for CONN analyses, maintaining a consistent sample size (n=24). CONN Functional Connectivity Toolbox (19c) (Whitfield-Gabrieli and Nieto-Castanon, 2012) was used to assess functional connectivity changes during the 2-back task before and after the one-month low glutamate diet. The same outlier criterion was used for CONN analyses, maintaining a consistent sample size (n=24).

The default preprocessing pipeline was used, which included: functional realignment and unwarping (Andersson et al., 2001); slice-timing correction (Henson et al., 1999); outlier identification; direct segmentation (i.e., grey matter, white matter, and CSF) and normalization into standard MNI space (Ashburner and Friston, 1997); and functional smoothing with a Gaussian kernel of 8mm full width half maximum.

Next, the default denoising pipeline in CONN was employed to reduce residual noise factors (e.g., physiological noise, outlier volumes, and motion effects) which can have considerable effects on functional connectivity analyses. Linear regression was used to estimate and remove confounding effects from each voxel, subject, and scanning session with anatomical component-based noise correction procedure, including noise estimates from white matter and CSF (Behzadi et al., 2007), 12 noise components (3 translation, 3 rotation, and their first-order derivatives) (Friston et al., 1996), identified outlier scans or scrubbing (Power et al., 2014), constant and first-order linear session effects, and constant task effects (Whitfield-Gabrieli and Nieto-Castanon, 2012). Temporal band-pass filtering was also used during the denoising pipeline (0.008 Hz – 0.09 Hz) (Hallquist et al., 2013). The effects of denoising were quality-checked by estimating the distribution of functional connectivity values across all participants before and after the low glutamate diet, and all functional connectivity values were normally distributed across both scanning sessions.

**Supplementary Table 1. Demographics and Measures of Memory and Cognition**

| **N = 24** | **Males**  **N (%)** | **Females**  **N (%)** |  |  |
| --- | --- | --- | --- | --- |
| Sex | 18 (75%) | 6 (25%) |  |  |
|  | **Baseline**  **Median (IQR)** | **Post-Diet**  **Median (IQR)** | ***p-value**** | ***Cohen’s d*** |
| Age (years) | 53.33 (5.85) | N/A | N/A | N/A |
| Weight | 209.00 (75.00) | 206.75 (67.00) | 0.012 | 0.03 |
| BMI | 31.09 (8.00) | 30.65 (7.11) | 0.016 | 0.06 |
| NCI | 95.00 (27.00) | 100.00 (24.50) | 0.037 | 0.19 |
|  | **Baseline**  **Mean (SD)** | **Post-Diet**  **Mean (SD)** | ***p-value***** | ***Cohen’s d*** |
| FFQ | 71.13 (18.60) | 6.50 (6.00) | <0.001 | 4.68 |
| Symptom Score | 20.29 (6.08) | 11.5 (5.76) | <0.001 | 1.48 |
| Memory (Composite) | 87.33 (18.74) | 89.42 (22.42) | 0.460 | 0.10 |
| Verbal Memory | 81.83 (19.30) | 87.29 (22.44) | 0.046 | 0.26 |
| Visual Memory | 98.13 (15.18) | 96.04 (16.51) | 0.525 | 0.13 |
| Executive Function | 93.33 (17.71) | 97.79 (20.06) | 0.160 | 0.23 |

FFQ= Food Frequency Questionnaire, NCI= Neurocognitive Index *Wilcoxon signed-rank test

** Paired t-test

**Supplementary Table 2. Clusters from Whole-Brain BOLD Signal Affected by the 1-Month Low Glutamate Diet**

| **Main Region** | **Additional Region** | **MNI Coordinates**  **(X, Y, Z)** | **K** | **T** | **p-uncorr** | **Cohen’s**  ***d*** | **Network** |
| --- | --- | --- | --- | --- | --- | --- | --- |
| L Superior Orbitofrontal Cortex | L Superior Medial Frontal Cortex | -16 +60 -2 | 40 | 4.13 | <0.0001 | 1.72 | DMN |
| L Superior Medial Frontal Cortex |  | -12 +64 +6 | 40 | 3.37 | <0.001 | 1.41 | DMN |
| R Crus II |  | +38 -76 -42 | 55 | 3.97 | <0.0001 | 1.66 | DMN |
| R Middle Frontal Cortex | R Superior Frontal Cortex | +26 +58 +24 | 47 | 3.91 | <0.0001 | 1.63 | FPN |
| R Middle Frontal Cortex | R Superior Frontal Cortex | +26 +44 0 | 32 | 3.57 | <0.001 | 1.49 | FPN |
| R Middle Frontal Cortex | R Superior Frontal Cortex | +28 +56 +8 | 32 | 3.08 | <0.003 | 1.28 | FPN |
| L Inferior Frontal Gyrus, pars triangularis |  | -48 +28 +12 | 49 | 3.43 | <0.001 | 1.43 | FPN |
| L Inferior Frontal Gyrus, pars triangularis | L Middle Frontal Gyrus | -38 +38 +14 | 49 | 3.16 | <0.002 | 1.32 | FPN |

Baseline _2-back > 0-back_ > Post-diet _2-back > 0-back_. Cluster defining threshold p<0.005 (uncorrected), cluster size (k) > 25. Network = cortical and cerebellar networks from Yeo et al. 2011 and Buckner et al. 2011 7-network parcellations. DMN = Default mode network. FPN = Frontoparietal network.

**
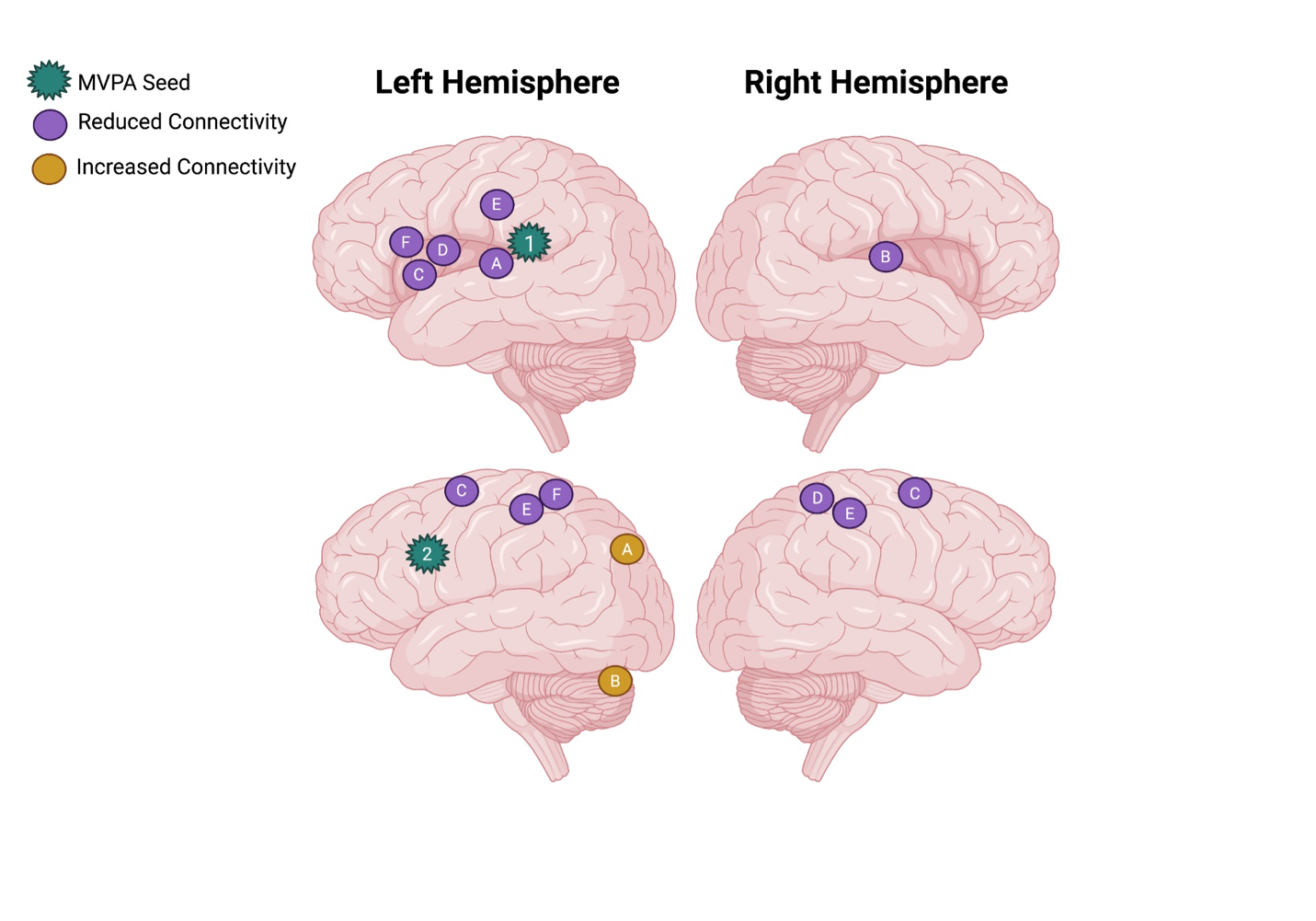
**

**Supplementary Figure 2.** Overview of Task-Based Functional Connectivity Changes Associated with the 1-Month Low Glutamate Diet. The green bursts indicate the MVPA-derived seeds, while the purple circles indicate regions of decreased connectivity, and the yellow circles indicate areas of increased activity. The letters within the circle correspond with order in Table 4. The MVPA-derived seed 3 did not have any significant connectivity changes post-diet and is therefore not shown.


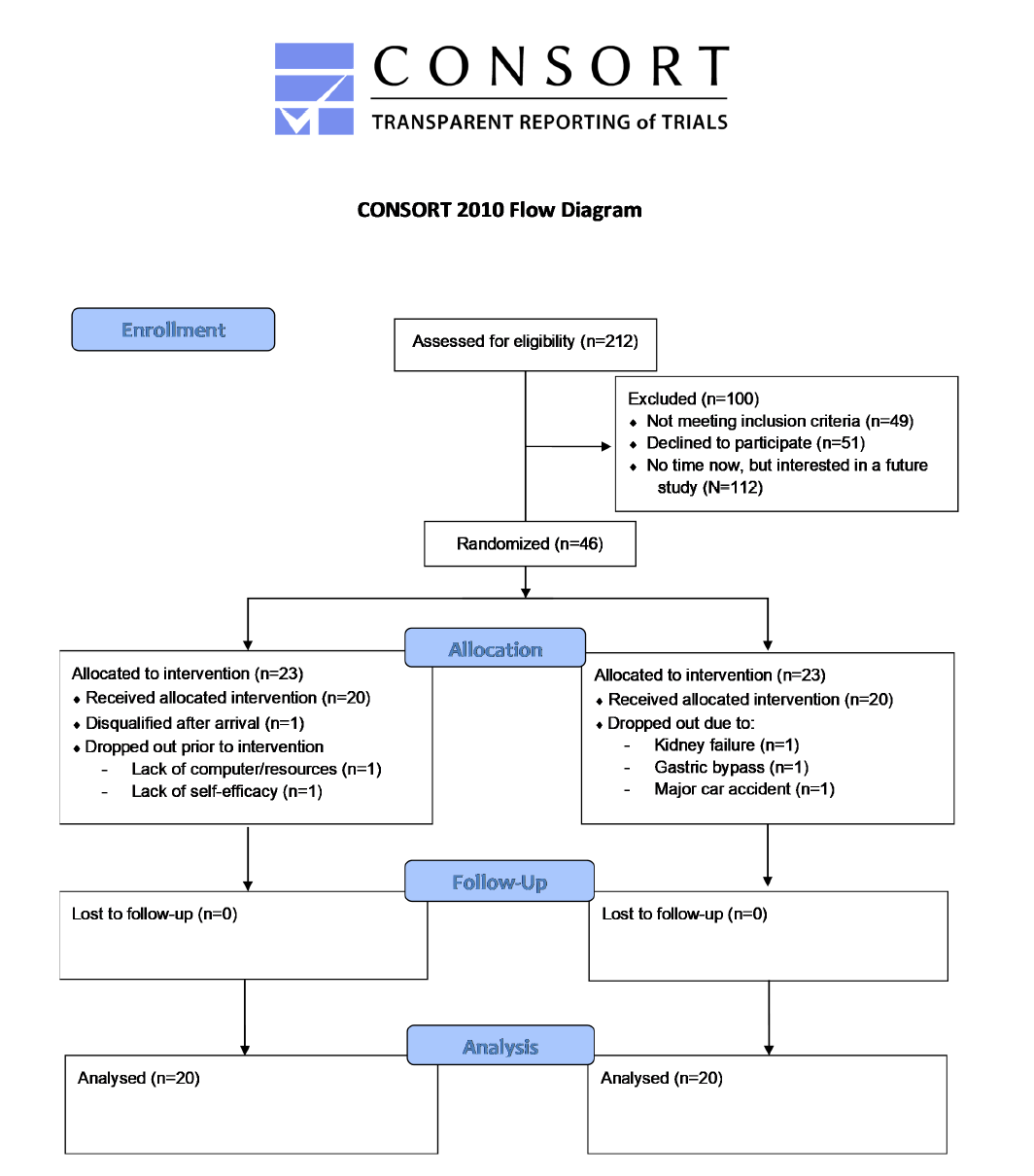


**Supplementary Figure 3.** CONSORT diagram of full placebo-controlled crossover randomized trial. Adapted from: Holton, K. F., Kirkland, A. E., Baron, M., Ramachandra, S. S., Langan, M. T., Brandley, E. T., & Baraniuk, J. N. (2020). The Low glutamate diet effectively improves pain and other symptoms of gulf War illness. *Nutrients,* 12(9), 2593.
